# Physiological and Pathogenic Roles of Prolyl Isomerase Pin1 in Metabolic Regulations via Multiple Signal Transduction Pathway Modulations

**DOI:** 10.3390/ijms17091495

**Published:** 2016-09-07

**Authors:** Yusuke Nakatsu, Yasuka Matsunaga, Takeshi Yamamotoya, Koji Ueda, Yuki Inoue, Keiichi Mori, Hideyuki Sakoda, Midori Fujishiro, Hiraku Ono, Akifumi Kushiyama, Tomoichiro Asano

**Affiliations:** 1 Department of Medical Science, Graduate School of Medicine, University of Hiroshima, 1-2-3 Kasumi, Minami-ku, Hiroshima City, Hiroshima 734-8551, Japan; nakatsu@hiroshima-u.ac.jp (Y.N.); ymatsunaga@hiroshima-u.ac.jp (Y.M.); ymmty@hiroshima-u.ac.jp (T.Y.); urouedakouji@yahoo.co.jp (K.U.); m164596@hiroshima-u.ac.jp (Y.I.); morikeiichi2@gmail.com (K.M.); 2 Division of Neurology, Respirology, Endocrinology and Metabolism, Department of Internal Medicine, Faculty of Medicine, University of Miyazaki, 5200 Kihara, Kiyotake, Miyazaki 889-1692, Japan; hideyuki_sakoda@med.miyazaki-u.ac.jp; 3 Division of Diabetes and Metabolic Diseases, Nihon University School of Medicine, Itabashi, Tokyo 173-8610, Japan; midori.fujishiro@gmail.com; 4 Department of Endocrinology and Diabetes, School of Medicine, Saitama Medical University, Moroyama, Saitama 350-0495, Japan; hono@saitama-med.ac.jp; 5 Division of Diabetes and Metabolism, The Institute for Adult Diseases, Asahi Life Foundation, Chuo-ku, Tokyo 103-0002, Japan; a-kushiyama@asahi-life.or.jp

**Keywords:** Pin1, glucose metabolism, lipid metabolism, vascular inflammation, bone formation

## Abstract

Prolyl isomerases are divided into three groups, the FKBP family, Cyclophilin and the Parvulin family (Pin1 and Par14). Among these isomerases, Pin1 is a unique prolyl isomerase binding to the motif including pSer/pThr-Pro that is phosphorylated by kinases. Once bound, Pin1 modulates the enzymatic activity, protein stability or subcellular localization of target proteins by changing the *cis*- and *trans*-formations of proline. Several studies have examined the roles of Pin1 in the pathogenesis of cancers and Alzheimer’s disease. On the other hand, recent studies have newly demonstrated Pin1 to be involved in regulating glucose and lipid metabolism. Interestingly, while Pin1 expression is markedly increased by high-fat diet feeding, Pin1 KO mice are resistant to diet-induced obesity, non-alcoholic steatohepatitis and diabetic vascular dysfunction. These phenomena result from the binding of Pin1 to several key factors regulating metabolic functions, which include insulin receptor substrate-1, AMPK, Crtc2 and NF-κB p65. In this review, we focus on recent advances in elucidating the physiological roles of Pin1 as well as the pathogenesis of disorders involving this isomerase, from the viewpoint of the relationships between signal transductions and metabolic functions.

## 1. Introduction

At present, prolyl isomerases (PPIases) are divided into three distinct groups, the FKBP (FK506 binding protein) family, Cyclophilin and the Parvulin family (Peptidylprolyl cis/tran*s* isomerase NIMA-interacting 1 (Pin1) and Par14) [1,2,3]. Among these isomerases, FKBPs and Cyclophilin have attracted attention as the primary binding partners of the immunosuppressive reagents rapamycin and cyclosporine A [4,5,6]. Pin1, however, was discovered in 1996 as a protein associating with NIMA which regulates mitosis [7].

Most peptide bonds are trans-form, while some of the peptide bonds of X-Pro can adopt the *cis*-conformation because of its unique stereochemistry [8,9,10]. All prolyl isomerases can catalyze *cis*-*trans* isomerization of the peptide bond between the preceding amino acid and proline [1,11]. Conformational changes of the substrate mediated by PPIase have significant impacts on enzyme activity, protein stability and localization [2,3,4,12].

**Figure 1:**
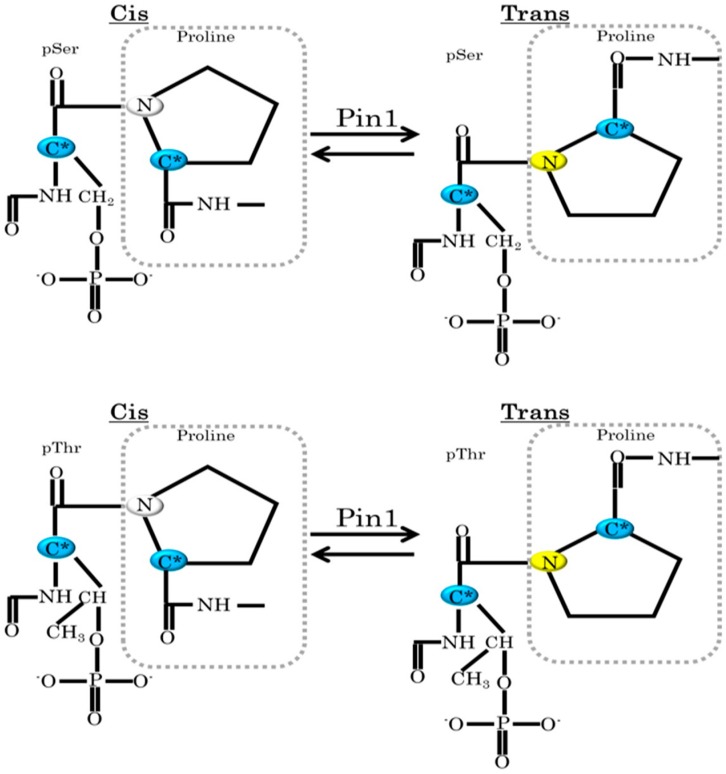
Pin1 recognizes the motif including pSer/pThr-Pro and catalyzes substrates. C*: asymmetric carbon.

Among prolyl isomerases, Pin1 is uniquely characterized by the requirement that Serine (Ser) or Threonine (Thr), as the amino acid preceding proline, be phosphorylated in order for direct binding with the substrate to take place [13] (Figure 1), while Par14 which belongs to the Parvulin family has low affinity for the pSer/pThr-Pro motif [14,15]. Interestingly, some protein kinases constitute the proline-directed protein kinase family, which includes the mitogen-activated protein kinase (MAPK) family, cyclin dependent kinases (CDKs), and Dual-specificity Tyrosine-phosphorylation Regulated Protein Kinase (DYRK) [16,17,18,19,20]. These kinases are responsible for cellular functions such as mitosis, differentiation and metabolism, as well as the onsets of various diseases, including malignancies, neurodegenerative disorders and metabolic syndromes [2,21,22,23]. The phosphorylation of substrates by these kinases and subsequent isomerization by Pin1 are essential for regulating multiple cellular functions [2,3]. Several studies have, in fact, revealed that Pin1-mediated isomerization plays important roles in various cellular functions [24,25,26]. In a relatively small number of cases, however, it is likely that Pin1 alters the functions of target proteins independently of its isomerase activities [27,28].

Since its discovery, Pin1 has become well established as a key factor in diverse cellular functions and the pathogenesis of a range of diseases. Several prior studies focused on the development of malignancies and Alzheimer’s disease, since Pin KO mice, especially the older animals, show abnormalities of cell proliferation and Tau accumulation [29,30]. In various malignancies, Pin1 expression levels are increased, and Pin1 gene silencing or knockdown suppresses cancer cell proliferation and migration [31,32,33]. In addition, older Pin1 KO mice show both a behavior disorder and tau accumulation in the brain, indicating Pin1 deficiency to be a risk factor for Alzheimer’s disease [24,30]. Thus, while it may appear as if Pin1 deficiency exerts opposite effects on two representative age-related diseases, this is actually due to the differences in the features of neuronal versus cancer cells. Since Pin1 promotes cell proliferation, dysfunctions involving mitosis due to Pin1 deficiency significantly impact the growth of malignant cells. On the other hand, Pin1 functions to enhance the survival of neurons, while being unable to promote the proliferation of mature neurons [34]. Pin1, however, plays important roles in enhancing the degradations of Tau and regulating the processing of amyloid beta precursor [24,35]. Therefore, Pin1 deficiency causes neurodegenerative disorders via Tau accumulation and amyloid beta.

Furthermore, Harris and colleagues have discussed the causes of these opposite effects of Pin1 on malignant tissues versus neurons, focusing on the differences in metabolic regulation between them [36]. Pin1 associates with both pyruvate kinase isoform M2 (PKM2) and phosphoglycerate kinase 1 (PGK1) in some malignances, and enhances nuclear translocation or mitochondrial translocation, leading to the Warburg effect and a decrease in reactive oxygen species (ROS) generation from mitochondria [37,38]. On the other hand, Pin1 dysfunction reduces glycolysis, but increases metabolism through oxidative phosphorylation. Amyloid beta enhances ROS generation from mitochondria during respiration, such that Pin1 dysregulation induces the accumulation of amyloid beta and a switch to oxidative phosphorylation, leading to Alzheimer’s disease.

Recent studies including ours have obtained evidence indicating that Pin1 is involved in a wide range of diseases, such as diabetes, non-alcoholic steatohepatitis (NASH), obesity, osteoporosis and cardiac hypertrophy. In this review, we focus on the role of Pin1 in various metabolic disorders, including the components of metabolic syndrome.

## 2. Pin1 Functions Are Regulated by Protein Kinases and SUMOylation

**Figure 2:**
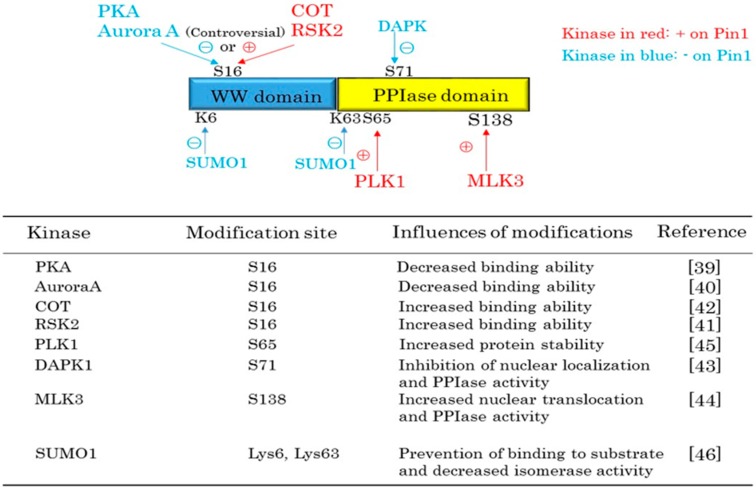
Pin1 functions are regulated by post-translational modifications. Some kinases phosphorylate Pin1 at Ser16 and may change the affinity of Pin1 for its substrates. However, the latter idea remains controversial. Pin1 phosphorylation by PLK1 and MLK3 upregulates the functions of Pin1 (shown as ⊕), while modifications by DAPK1 or SUMO1 downregulate Pin1 functions (shown as ⊝).

The interaction between Pin1 and substrates is strictly controlled by protein kinases. First, the phosphorylation of substrates on the Ser/Thr-Pro motif by kinases is essential for Pin1 binding [13]. Second, the functions of Pin1 itself are controlled by kinases (Figure 2). The first report on Pin1 modification described phosphorylation in the WW domain, which is responsible for binding to the pSer/pThr-Pro motif in substrates. Some kinases have been suggested to have the ability to phosphorylate Ser16 in the WW domain and thereby change binding affinities for its substrates, but whether this modification exerts such effects remains controversial. Lu et al. reported that phosphorylation at Ser16 by PKA can inhibit the association between Pin1 and its substrate, MPM2-antigen [39]. Ser16 in the WW domain is also reportedly phosphorylated by aurora A, which eliminates the binding capacity of Pin1 [40]. In contrast, it was reported that RSK2 or COT induces Pin1 phosphorylation at the Ser 16 site, which increases the binding capacity of Pin1 to its substrates [41,42]. These discrepant observations might be explained by the possibility that these kinases can phosphorylate Pin1 at other sites and thereby alter binding to other substrates. Taken together, current findings suggest that Pin1 phosphorylation at the Ser16 site changes the binding ability of Pin1, depending on its substrate. In addition, death-associated protein kinase 1 (DAPK1) phosphorylates Pin1 on Ser71 in the PPIase domain, and thereby inhibits nuclear translocation and catalytic activity [43], while phosphorylation of Pin1 on Ser138 by mixed-lineage kinase 3 (MLK3) enhances both [44]. Polo-like kinase 1 (PLK1) can also phosphorylate Pin1 at the Ser65 site, thereby increasing the stability of Pin1 protein by inhibiting its ubiquitination, though phosphorylation of the Ser65 site has no effect on Pin1 activity [45]. Finally, SUMOylation of Pin1 at Lys6 and Lys63 by SUMO1 prevents Pin1 from binding to its substrates and downregulates isomerase activity [46].

These post-translational modifications of Pin1 are well known to correlate with cancer progression, but it is unclear whether these alterations contribute to the development of other diseases, such as metabolic syndromes. However, for example, MLK3 is reportedly involved in steatohepatitis developing in response to the feeding of a high-fat high-carbohydrate diet [47]. Although the authors describe JNK activation by MLK3 as contributing to steatohepatitis progression, positive regulation of Pin1 by MLK3 may also be involved because Pin1 enhances the formation of hepatic steatosis as described in more detail below. Thus, as with cancers, modifications of Pin1 may well contribute to the development of metabolic syndromes.

## 3. Pin1 Is Highly Involved in the Development of Metabolic Syndrome

Although Pin1 expressions are well known to be markedly upregulated in different types of cancers [33], whether Pin1 protein levels are altered in metabolic syndromes remains unknown. Interestingly, we have found high fat diet (HFD) feeding to dramatically increase Pin1 expression levels in the liver, muscle and white adipose tissues (WAT) [26]. HFD feeding induces the onset of obesity, nonalcoholic fatty liver disease (NAFLD) and diabetes mellitus, all of which are features observed in the metabolic syndrome. NASH is characterized by not only lipid accumulation but also inflammation in the liver. Increased Pin1 protein expression was observed in the livers of mice with NAFLD induced by HFD, as well as in the livers of mice with NASH induced by feeding of a methionine-choline deficient diet (MCDD) [48]. These lines of evidence led us to consider the possibility of a close relationship between metabolic syndrome and increased Pin1 expression. Hyperglycemia and atherosclerosis are also among the phenotypes of the metabolic syndrome. In fact, interestingly, recent studies have revealed that Pin1 expression is increased in the aortas of mice with streptozotocin (STZ)-induced hyperglycemia as well as in the arterial walls of those treated with serum from diabetic mice [49,50]. In addition, increased Pin1 expression is observed in the heart after transaortic construction (TAC) [51].

Importantly, it is likely that the changes in Pin1 expression take place prior to the development of physiological dysfunctions. For example, Pin1 expressions are increased one week after HFD feeding in WAT. However, the differences in body weight between wild and Pin1 KO mice are detected after four weeks. Moreover, TAC induced the upregulation of Pin1 expressions after two weeks, while the cardiac function abnormalities, such as fractional shortening, only became evident after four weeks. Thus, the increased Pin1 expressions are considered to be causative for, rather than resulting from, various phenotypes among those associated with the metabolic syndrome.

**Figure 3:**
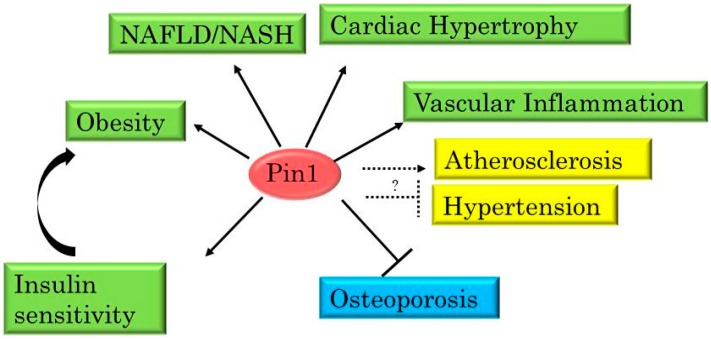
Pin1 plays important roles in the development of diseases. Pin1 promotes the progression of obesity, cardiac hypertrophy, vascular inflammation and NASH (shown as solid arrows), while suppressing osteoporosis. Pin1 also increases insulin sensitivity in metabolic organs, but this enhancement may be linked to obesity. The roles of Pin1 in atherosclerosis and hypertension are unresolved issues (shown as dotted lines and question mark).

Altered Pin1 expression is also observed during cell differentiation. For example, elevated Pin1 expressions are observed during adipogenesis in 3T3-L1 adipocyte cells and in dental pulp stem cells [26,52,53]. Magli et al. showed the distribution of Pin1 protein to be altered during muscle differentiation [54]. Thus, Pin1 expressions in metabolic organs show change, depending on nutritional status. However, several aspects of the regulation of Pin1 expression remain unclear and are expected to be resolved by future investigations. We suspect that Pin1 alterations in some organs take part in the regulation of metabolic functions or even the onsets of metabolic syndromes, and we have thus been studying the roles of Pin1 in metabolic functions (Figure 3). Indeed, we and other research groups have demonstrated that Pin1 contributes to glucose metabolism, the maintenance of vascular functions and bone formation, as it does to the development of metabolic syndromes [26,48,49,52,53]. In addition, treatment with a Pin1 inhibitor can reportedly ameliorate diabetic vascular dysfunctions [49,50,55].

Finally, Pin1 expressions in serum might serve as disease markers because some investigators have indicated that altered Pin1 expressions are detectable in patients suffering from metabolic syndromes. Cengis et al. investigated whether serum Pin1 would be useful as a biomarker of NASH and found serum Pin1 levels to be significantly higher in a group with severe fibrosis than in a group with mild fibrosis, leading to the conclusion that Pin1 is a potential marker of NASH [56]. Paneni et al. reported both the expression and the activity of Pin1 to be elevated in the peripheral blood monocytes of diabetic patients and to correlate with both Hb1Ac and fasting plasma glucose [50].

Taking these results together, changes in Pin1 that depend on nutritional status appear to be tightly linked to metabolic disorders and Pin1 may thus be a useful biomarker. In addition, a Pin1 inhibitor or modulators of proteins upstream from Pin1 might be promising targets for treating metabolic syndromes including diabetic vascular dysfunctions.

## 4. Pin1 Controls Insulin Signaling

Insulin signaling regulates glucose uptake, lipid biosynthesis and cellular growth in insulin sensitive tissues, and dysfunction of this pathway drives the development of type 2 diabetes, NASH, atherosclerosis and obesity. Accordingly, many investigations have been undertaken to discover mechanisms which impair or enhance insulin signaling, and certain key proteins, such as insulin receptor substrate (IRS), phosphatidyl-inositol-3-kinase (PI3-kinase) and Akt have thereby been identified [57,58].

We initially identified Pin1 as an IRS-1 binding partner and reported that Pin1 enhances insulin signaling in certain tissues [26]. Pin1 overexpression in HepG2 was found to enhance tyrosine phosphorylation of IRS-1, binding with the p85 subunit and Akt activation, while knockdown of Pin1 suppressed these activities. As the inactive form of PPIase was shown to have no effect on tyrosine phosphorylation of IRS-1, prolyl isomerization of IRS-1 by Pin1 was assumed to be essential for the regulation of IRS-1. Although the mechanism is unclear, we suggest that isomerization by Pin1 facilitates IRS-1 contact with insulin receptors responsible for the tyrosine phosphorylation of IRSs. Another possibility is that the protein tyrosine phosphatase which dephosphorylates IRSs might be blocked from accessing the isomerized-IRS-1.

**Figure 4:**
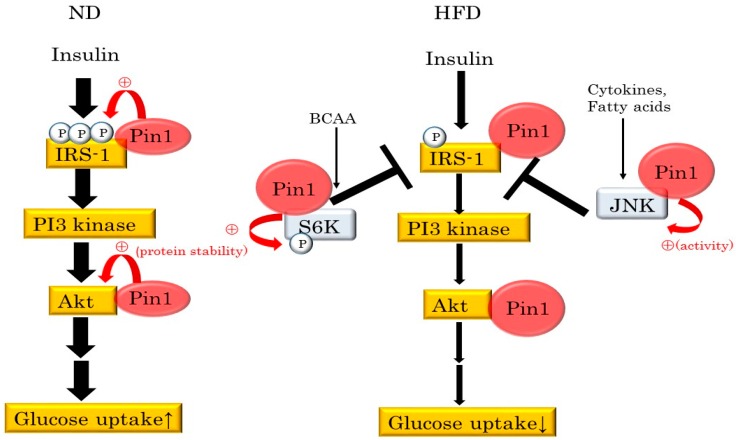
Pin1 has biphasic effects on insulin signaling. Pin1 associates with IRS-1 and enhances insulin signaling through upregulation of tyrosine phosphorylation in animals fed normal diets or under lean conditions. Additionally, Pin1 binds to JNK and S6K, thereby triggering insulin resistance, and both are activated by HFD feeding. Pin1 also increases the kinase activities of JNK and S6K. Positive effects exerted by Pin1 on IRS-1 can be nullified by both JNK and S6K under HFD feeding conditions, despite Pin1 expressions being increased. (P): phosphorylation of substrates; (+): Pin1 enhances phosphorylation, activity and protein stability; (T): Both JNK and S6K downregulates tyrosine phosphorylation of IRS-1. (↑) Glucose is effectively incorporated into the tissues; (↓) Glucose uptake is impaired.

In addition, Pin KO mice show abnormalities of glucose tolerance and insulin signaling in both liver and muscle. Thus, Pin1 promotes insulin signaling via IRS-1 phosphorylation. Interestingly, Pin1 has no effect on IRS-2 phosphorylation, although Pin1 binds to IRS-2. However, Pin1 also associates with c-Jun N-terminal kinase (JNK) or S6 kinase (S6K), triggering insulin resistance through serine phosphorylation of IRSs [59,60]. Pin1 binding and the subsequent isomerization enhance stress-induced JNK activation and S6K phosphorylation. Taken together, these findings indicate that the increases in both Pin1 and stressors such as cytokines, in response to HFD feeding, lead to hyper-activation of JNK or S6K. Consequently, HFD feeding is considered to cancel the positive effect of Pin1 on IRS-1, instead causing insulin resistance (Figure 4).

## 5. Pin1 Suppresses Gluconeogenesis

In the fasted state, hepatic gluconeogenesis plays an important role in maintaining glucose balance. Gluconeogenesis is tightly regulated by the expression levels of rate-limiting enzymes, specifically phosphoenolpyruvate carboxykinase (PEPCK) and glucose 6-phosphatase (G6Pase), which are controlled by transcription factor cAMP response element binding protein (CREB) and two coactivators, the transcriptional co-factors including CREB binding protein (CBP) and creb-regulated transcriptional co-factor2 (CRTC2) [61,62,63]. During the fasting period, circulating glucagon triggers gluconeogenesis via the cAMP-Protein Kinase A (PKA) pathway. Activated PKA mediates CREB phosphorylation, which permits recruitment of CBP and thereby increases transcriptional activity. At the same time, PKA promotes the dephosphorylation of CRTC2, with its consequent translocation from the cytosol to the nucleus where CRTC2 interacts with CREB, thereby upregulating CREB activity.

Our search for Pin1 binding proteins yielded CRTC2 [64]. Pin1 overexpression suppresses, while Pin1 knockdown enhances, CRE-transcriptional activity. Pin1 inhibits the shuttling of CRTC2 to the nucleus. Moreover, in STZ-treated mice, Pin1 overexpression in the liver improves hyperglycemia and suppresses increases in PEPCK and G6Pase. Another study showed that Pin1 associates with CREB and suppresses its activity, in an isomerase-dependent fashion [65].

However, hepatic signal transducer and activator of transcription 3 (Stat3) is known to suppress gene expressions of gluconeogenic enzymes [66,67]. Lufei et al. showed that Pin1 associates with Stat3 and enhances IL-6-induced transcriptional activity in HepG2 cells, independently of PPIase activity [28]. Their study revealed that Pin1 deficiency impairs the DNA binding ability of Stat3, but without altering the levels of nuclear translocation. Although the authors provided no description of the relationship between Pin1-Stat3 and gluconeogenesis, we suggest that Pin1 can suppress gluconeogenesis through Stat3 activation. These observations, taken together, indicate that Pin1 works as a negative regulator of gluconeogenesis by suppressing the CRTC2-CBP-CREB pathway and enhancing the IL6-Stat3 pathway.

## 6. Pin1 Is Essential for Adipogenesis and Is Involved in Obesity

Obesity results from not only excessive food intake but also insufficient energy expenditure. In adipose tissues, insulin signaling facilitates lipid synthesis by inducing the rate-limiting enzymes, such as acetyl Coa carboxylase 1 (ACC1) or fatty acid synthase (FASN), thereby leading to adipogenesis [68]. Indeed, deletion of the Pin1 gene or Pin1 inhibitor treatment reduces the accumulation of lipid droplets and the expressions of mature adipocyte markers in both 3T3-L1 and MEF cells [26,65]. Consistent with the in vitro data, Pin1 KO mice show resistance to HFD-induced obesity [26]. Body weight, adipocyte weight and lipid droplet sizes are reduced in Pin1 null mice [65]. Gene expressions of enzymes involved in fatty acid synthesis are also reduced in white adipose tissues of Pin1 KO mice [26]. Moreover, a recent study demonstrated that Pin1 associates with PPARγ, which is a master regulator of adipogenesis and enhances the transcriptional activity of PPARγ, thereby leading to adipogenesis [53]. As another mechanism, Jiang et al. described Pin1 expressions as being altered during mitotic clonal expansion [69]. Their study revealed PKM2, which associates with Pin1, to be essential for mitotic clonal expansion and adipogenesis. However, the details of the underlying mechanism remain unclear. Therefore, Pin1 might be essential for mitotic clonal expansion which is a first step in adipogenesis, governed at least in part through the regulation of PKM2. This hypothesis is supported by the observation that Pin1 inhibition or knockdown suppresses, while Pin1 overexpression increases, C/EBPβ expression, induced in the early stage of adipogenesis [26,52]. Accordingly, Pin1 is an essential factor in adipogenesis, although the underlying mechanistic details have yet to be clarified.

Muscle is one of the tissues that consumes energy through fatty acid oxidation AMP-activated protein kinase (AMPK) has been revealed to regulate energy homeostasis in the hypothalamus, liver and muscle [70,71]. AMPK is composed of three subunits, the α subunit serving as a catalytic subunit, and the β and γ subunits which have regulatory functions [69]. The phosphorylation of T172 in the α subunit is essential for AMPK activation, and LKB1 or CamKK plays this role as an AMPK kinase [72,73]. In addition, the binding of 5’-AMP with the γ subunit inhibits dephosphorylation of the α subunit by PP2C and promotes the association between LKB1 and the α subunit [74,75]. AMPK is well known to indirectly upregulate fatty acid oxidation and AMPK activators, such as metformin, have been used for treating metabolic syndrome [71,76].

Phosphorylation of the α subunit in AMPK is also regulated by Pin1 [77]. Pin1 overexpression was shown to remarkably decrease, while Pin1 knockdown enhanced, AMPK phosphorylation by 2-deoxy-glucose (2-DG). Interestingly, it was found that Pin1 associates with the γ subunit of AMPK. The AMPK γ subunit possesses four CBS domains and is thus able to bind to 5′-AMP. The Pin1 binding site (T211-Pro) in the AMPKγ1 subunit exists in the CBS3 domain, suggesting the positive effect of 5′-AMP on AMPK to be strongly influenced by Pin1.

This suggestion is well supported by the observation that Pin1 has no impact on phosphorylation or dephosphorylation of AMPK without 5′-AMP, but Pin1 cancels the positive effects gained by the addition of 5′-AMP. These results suggest that Pin1 disrupts either the binding of 5′-AMP to the CBS domain or the conformational change induced by 5′-AMP. In addition, Pin1 KO mice show enhanced AMPK activation as well as elevated expressions of mitochondria-related genes, associated with reduced triglyceride deposition in muscle.

Interestingly, Pin1 reportedly takes part in muscle differentiation [54]. Pin1 associates with MEF2C, a critical regulator of myogenesis, and decreases its stability, leading to suppression of muscle differentiation.

These findings, collectively, indicate that Pin1 promotes obesity by increasing lipid synthesis in adipose tissues and reducing fatty acid oxidation in muscle. Pin1-mediated fatty acid accumulation may be linked to cancer progression because cancer cells require an energy source for proliferation. Pin1 is thus a potential target for treatments of obesity and sarcopenia as well as cancers.

## 7. Pin1 Plays a Critical Role in the Development of NASH

NASH is characterized by lipid accumulation, inflammation and fibrosis in the liver [78]. We found that Pin1 deficiency can prevent the onset of MCDD-induced NASH featuring hepatic steatosis, inflammation and fibrosis [49]. As described above, Pin1 promotes fatty acid accumulation through multiple mechanisms. In addition, Pin1 reportedly promotes fibrosis through the TGF-Smad pathway [79,80,81]. Pin1 associates with Smad2/3, and enhances its phosphorylation and subsequent transcriptional activity. Pin1 deficiency or Pin1 inhibitor treatment reduces Smad phosphorylation and the expression of PAI-1, which is induced by the TGF-Smad pathway. Furthermore, the expression of CTGF is also diminished in Pin1 KO mice [48]. In the liver, Stat3 reportedly takes part in CTGF induction [82]. Since Pin1 enhances Stat3 transcriptional activity, we speculate that Pin1 induces CTGF expression through the Stat3 pathway.

To date, there are no reports describing Pin1 inhibitors as possibly ameliorating NASH pathology.

However, treatment with the Pin1 inhibitor juglone was reported to ameliorate drug-induced liver fibrosis [80]. Therefore, the development of Pin1 inhibitors which can alleviate the pathology of NASH is highly anticipated.

## 8. The Role of Pin1 in Hypertension

Hypertension is one of the most common features of the metabolic syndrome. The contraction and extension of blood vessels are regulated by various factors [83]. Nitric oxide synthase (NOS) plays an important role in the dilation of blood vessels. Numerous reports have demonstrated hypertension to be related to impairment of the NOS-NO cascade [84,85]. It is well known that the activity of endothelial NOS (eNOS) is regulated by serine and threonine phosphorylations at multiple sites. Phosphorylation of eNOS by Akt or AMPK enhances both eNOS activity and the production of NO [86,87], while CDK5 or ERK reduces both via phosphorylation of the Ser116 site [88,89].

There are also studies providing evidence that Pin1 regulates NO production and vascular functions, but further investigation is necessary to fully elucidate the role of Pin1 in vascular maintenance.

Chiasson et al. described Pin1 as associating with eNOS and Pin1 knockdown as promoting eNOS phosphorylation at Ser116, thereby decreasing the activity of eNOS in rat aortic endothelial cells [90]. In addition, the acetylcholine-induced relaxation response is decreased in aortic specimens from Pin1 KO mice, as compared to those from WT mice. The authors insisted that Pin1 plays a positive role in the maintenance of vascular functions. Additionally, Cho et al. reported that Pin1 knockdown by siRNA attenuates lipopolysaccharide-induced NO production [91].

However, Ruan et al. reported that Pin1 is a negative regulator of NO production in bovine aortic endothelial cells [92]. They showed that Pin1 overexpression reduces, while Pin1 inhibiter treatment increases, NO production. Other groups have obtained data supporting the results of Ruan and colleagues. Kennard et al. argued that TNE treatment decreases NO production through binding of eNOS with Pin1 [93]. They showed overexpression of dominant-negative Pin1 or treatment with the Pin1 inhibitor Juglone to restore downregulation of NO production by TNF in bovine aortic endothelial cells. Paneni et al. demonstrated that high glucose inhibits NO release in human aortic endothelial cells via increased Pin1 expression [50]. Their study also revealed that aortic specimens from Pin1 KO mice show normal relaxation in response to acetylcholine, even under diabetic conditions.

These studies raise the possibility that the roles of Pin1 in NO production and hypertension are reciprocal. The inconsistencies among the results may be due to variable experimental conditions, such as using different cell lines, stimuli and animal models because other substrates also reportedly show reciprocal actions under the influence of Pin1 in different cell types [27,53,79,80,94]. In addition, in some experiments, Juglone is used as a Pin1 inhibitor. However, Juglone is a non-specific inhibitor because it associates with cysteine-rich proteins, such as tubulin, or induces the generation of ROS [95,96,97]. Therefore, the results obtained with juglone are not necessarily due to Pin1 inhibition and may account for the discrepant results described above. Wang et al. advocated that the involvement of iNOS be investigated because Pin1 regulates iNOS by promoting both its induction and its degradation [98]. Despite the discrepant results, it seems certain that Pin1 is involved in NO production in the aorta and we anticipate that elucidating of precise mechanism(s) will contribute to developing treatments for hypertension.

## 9. The Roles of Pin1 in Atherosclerosis and Cardiac Dysfunction

Atherosclerosis results from the interactions of various components of the metabolic syndrome, including diabetes, hypertension and hyperlipidemia [99]. Atherosclerosis is considered to be one of chronic inflammatory diseases and its progression is mediated by macrophages [100]. Macrophages release inflammatory cytokines and cells take up lipids, thereby becoming foam cells.

As yet, there have been no reports indicating Pin1 to be involved in the formation of atherosclerotic lesions in ApoE KO or LDL-R KO mice. However, some reports have shown that vascular inflammation is regulated by Pin1. Paneni et al. described Pin1 as upregulating high glucose-induced inflammatory adhesion molecules, such as vascular adhesion cell molecule-1 (VCAM-1), intercellular cell adhesion molecule 1 (ICAM-1) and MCP-1, through the NF-κB pathway [50]. Pin1 binds to the p65 subunit of NF-kB and increases its stabilization [101]. In macrophages, upregulation of p65 by Pin1 enhances the induction of inflammatory cytokines such as TNF [48,102]. In addition, Pin1 promotes the generation of ROS in neutrophils by regulating NADPH oxidase [103,104]. Thus, the increases in adhesion molecules and inflammatory cytokines induced by Pin1 coordinate chronic inflammation in endothelial cells, possibly leading to atherosclerosis.

Furthermore, Pin1 expressions are increased after TAC and Pin1 is involved in cardiac hypertrophy through regulating the Akt and Raf-MEK pathways [51]. Notably, Pin1 KO mice have preserved cardiac functions and are able to survive after exposure to TAC. Consistent with the in vivo data, Pin1 knockdown in cardiomyocytes decreases the phosphorylation of both Akt and MEK, and normalizes the cell size expansions in response to serum stimulation. Interestingly, cardiac specific Pin1 overexpression also prevents hypertrophy by suppressing the MEK pathway. Pin1 overexpression in cardiomyocytes induce Raf-1 phosphorylation which is a factor upstream from MEK, and attenuates kinase activities, thereby inhibiting the MEK pathway. Taken together, these observations indicate that Pin1 plays important roles in maintaining cardiac vascular functions and that Pin1 inhibitors might constitute a novel approach to preventing cardiovascular dysfunctions.

## 10. The Role of Pin1 in Bone Formation and Osteoporosis

Pin1 is reportedly a key regulator of bone formation. Bone is repeatedly broken down and restored via the actions of both osteoblasts and osteoclasts. Any imbalance between these two cell types causes bone disease, as exemplified by osteoporosis [105]. Osteoblast differentiation is controlled by both BMP (bone morphogenetic protein)-SMAD and Wnt-β Catenin signaling [106], while osteoclast differentiation is triggered by RANKL [107].

Interestingly, Pin1 KO mice were reported to have features similar to those of osteoporosis [108]. Shen et al. reported that Pin1 null mice show low bone mass and bone mineral density [109]. They describe Pin1 deficiency as attenuating osteoblast functions and bone formation, though Pin1 exerted no effect on osteoclast differentiation. Pin1 was found to interact with Smad1 and Smad5, and enhance BMP signaling [110]. In addition, Pin1 associates with Runx2 which is an essential transcriptional factor for osteoblast differentiation and is activated by BMP signaling [111]. Phosphorylation of Runx2 by ERK enables its binding with Pin1, and isomerized-Runx2 bound to Pin1 can be acetylated by histone acetyltransferase [112]. Thereby, the binding of Pin1 to Runx2 upregulates Runx2 stabilization by inhibiting its polyubiquitination, leading to increased transcriptional activity. In addition, Pin1 increases, while Pin1 silencing decreases, the reporter activities of osteoblast genes triggered by Runx2 overexpression [113]. Pin1 is also involved in the regulation of Osterix, which is an essential factor for osteoblast differentiation [114]. Pin1 upregulates Osterix protein levels by inhibiting its polyubiquitination, thereby enhancing transcriptional activity. In addition, the binding of Pin1 to β-catenin functions to attenuate protein degradation [115] and Pin1 reportedly enhances nuclear translocation [32].

Islam et al. on the other hand, demonstrated that Pin1 is a negative regulator of osteoclast fusion [116]. Pin1 decreases DC-STMAP which is a master regulator of osteoclast fusion. It is widely known that Pin1 KO mice have relatively large osteoclasts, as compared to those in wild type mice.

Collectively, findings obtained to date suggest that Pin1 increases both BMP and Wnt signaling, which are essential for osteoblast formation, and suppresses osteoclast fusion. Together, these actions promote osteogenesis. Although changes in Pin1 expression and function with age remain unclear, either a stabilizer of Pin1 protein or a Pin1 activator may be of use for treating osteoporosis.

## 11. Conclusions

**Figure 5:**
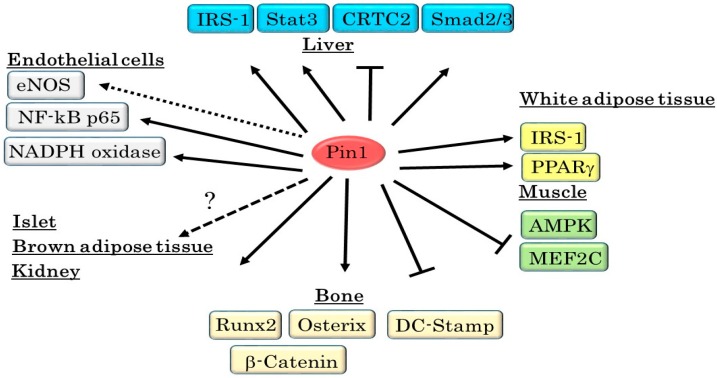
Pin1 regulates multiple signal transductions in metabolic organs. Pin1 enhances insulin signaling through binding to IRS-1 and suppresses gluconeogenesis by associating with CRTC2, CREB and Stat3 in the liver. In addition, Pin1 increases liver fibrosis through regulation of the TGF-Smad pathway. In adipocytes, upregulation of insulin signaling by Pin1 leads to the induction of lipid synthesis and obesity. However, Pin1 suppresses myogenesis via degradation of MEF2C and decreases fatty acid synthesis by inhibiting AMPK phosphorylation. Pin1 also enhances osteoblast formation and suppresses osteoclast fusion, thereby preventing osteoporosis. Furthermore, Pin1 is involved in vascular inflammation via the stabilization of p65 protein. Thus, while Pin1 clearly operates as a master regulator of metabolism, its roles in certain tissues, such as pancreatic islets, the kidney, and brown adipose, remain to be determined. (↑): Pin1 positively regulates functions of substrates; (T): Pin1 negatively controls functions of substrates; (dotted lines): The regulation by Pin1 have remained undetermined; (?): There are no reports which elucidate the roles of Pin1 in these tissues.

Pin1 is widely viewed as a master orchestrator of malignant transformation and tumor progression because Pin1 regulates numerous oncogenes and tumor suppressors. Metabolically, Pin1 modulates multiple signal transductions. Pin1 associates with and controls key molecules (IRS-1, CRTC2, AMPK, eNOS, Runx2 and others) involved in metabolic functions or the development of metabolic syndromes (Table 1). Upregulation of Pin1 in response to excessive nutrition has been suggested to cause energy deposition in cells or tissues. As clearly described herein, Pin1 is a master regulator of cellular energy metabolism (Figure 5), although the specific roles of Pin1 in pancreatic islets, the kidney, and brown adipose tissues remain to be elucidated.

We suspect that there are more Pin1 targeting proteins involved in the pathways governing metabolic functions. Therefore, future investigations should be designed to identify unknown targeting proteins and elucidate the precise mechanisms underlying metabolic pathway regulation by Pin1.

## Figures and Tables

**Table 1:** List of Pin1 substrates in metabolic regulation.

Substrate	Functional Changes Mediated By Pin1	Reference
IRS-1	Enhances Tyrosine Phosphorylation	[26]
CRTC2	Inhibits Nuclear Translocation	[64]
CREB	Suppresses Transcriptional Activity	[65]
Stat3	Upregulates Transcriptional Activity	[28]
AMPK	Decreases Phosphorylation of the α Subunit	[77]
PPARγ	Enhances Transcriptional Activity	[53]
Smad	Enhances Protein Stability and Phosphorylation	[79,80]
NF-κB p65	Increases Protein Stability	[101]
eNOS	Increases or Decreases eNOS Activity	[90,91,92,93]
Runx2	Enhances Transcriptional Activity	[111,112,113]
Osterix	Increases Protein Stability	[114]
β-Catenin	Increases Protein Stability	[115]
DC-STAMP	Decreases Protein Stability	[116]
